# Chinese Herbal Extractions for Relieving Radiation Induced Lung Injury: A Systematic Review and Meta-Analysis

**DOI:** 10.1155/2017/2141645

**Published:** 2017-03-29

**Authors:** Bo Deng, Chao Deng, Zhiqiang Cheng

**Affiliations:** ^1^Department of Oncology of Integrative Chinese and Western Medicine, China-Japan Friendship Hospital, Beijing 100029, China; ^2^Beijing University of Chinese Medicine, Beijing 100029, China

## Abstract

*Background*. Radiation induced lung injury (RILI) is one of the most common and severe side effects of thoracic radiotherapy. In this meta-analysis, the effects of Chinese herbal extractions (CHE) for preventing and treating RILI are evaluated.* Methods*. Randomized Controlled Trials (RCTs) from five databases were identified. Studies were evaluated and the relevant data were extracted by two authors independently. Differences were resolved by a third party. Meta-analysis was conducted using RevMan 5.0.* Results*. In total, 2734 participants receiving thoracic radiotherapy were included in 28 RCTs, and 16 CHE were evaluated. Meta-analysis showed that CHE intervention significantly reduced the incidence of acute radiation pneumonitis (RP) and radiation induced pulmonary fibrosis (RIPF). In CHE group, total effective rate and remission rate of RILI patients were significantly higher. Patient's quality of life (Qol) and clinical symptoms and signs were improved significantly. Inflammatory cytokines decreased, and thymus dependent lymphocytes subgroups were improved.* Conclusion*. CHE intervention may have clinical effectiveness for relieving RILI and related symptoms and signs and lead to improvement of Qol. However, more double-blind, multicenter, large-scale RCTs are needed to support this theory.* Trial Registration*. PROSPERO International prospective register of systematic reviews has registration number CRD42016043538.

## 1. Introduction

Thoracic radiation is an important curative and palliative treatment for lung cancer, esophagus cancer, breast cancer, mediastinal malignant tumors, and so on. Radiation induced lung injury (RILI) is one of the most common and severe side effects of thoracic radiotherapy [1]. RILI can occur in two phases, namely, early (<6 months) when it is called radiation pneumonitis (RP) and late (>6 months) when it is called radiation induced pulmonary fibrosis (RIPF) [1]. The reported rates of symptomatic RP after radiotherapy range from 9% to 51% [2–4]. Steroids and broad-spectrum antibiotics form the mainstay of treatment of RP. However their effectiveness is limited and their adverse effects are significant [1]. RP may trigger multiple repair mechanisms to restore lung function and remodel lung fibrosis; the incidence of RIPF varies from 8% to 43% [5, 6]. Unfortunately, there are no defined protocols or guidelines for the management of RIPF. Novel therapeutic approaches to improve the effectiveness of RILI treatment are required.

Chinese herbal medicine has been widely used alone or in combination with western conventional medicine to relieve RILI for more than 20 years. Multiple randomized clinical trials have suggested that Chinese herbal extractions (CHE) intervention can reduce symptoms and improve patient's quality of life and reduce proinflammatory cytokines and profibrosis cytokines [7–34]. In vivo, CHE can effectively relieve RP and RIPF, reducing proinflammatory cytokines (tumor necrosis factor-*α*, TNF-*α*) and profibrosis cytokines (transforming growth factor-*β*, TGF-*β*) in rat/mouse models of RILI [35–38]. However, there is no systematic review on effectiveness of CHE for relieving RILI. In this meta-analysis, the effectiveness and safety of CHE, for preventing and treating RILI, are evaluated for the first time.

## 2. Methods

### 2.1. Database and Search Strategies

Randomized Controlled Trials (RCTs) were identified by systematic searches in MEDLINE, Cochrane Controlled Trials, Springer, China National Knowledge Infrastructure (CNKI) database, and Wan-Fang database of China Science Periodical Database (CSPD) from the date of inception until April, 2016. The searching terms were “radiation induced lung injury/radiation pneumonitis/radiation induced pulmonary fibrosis” or “Traditional Chinese Medicine/herbal medicine” without language limitation. Reference lists from studies selected by electronic searching were hand searched.

### 2.2. Inclusion Criteria and Exclusion Criteria

We included trials with patients 18 years old and older, who have been clearly diagnosed with malignant tumors by pathology or cytology and were treated with thoracic radiotherapy. The dose of radiotherapy was not restricted. We ensured that patient age, gender, pathology, and disease stage were comparable among the treatment groups. CHE interventions should be approved by China Food and Drug Administration (CFDA), including single herb extractions and extractions from a compound of several herbs. The preparation method (e.g., oral decoction, powder, and injection) and delivery mode (e.g., oral, intravenous, and aerosol inhalation) of the CHE were not restricted. The control interventions were either placebo/no intervention, or conventional treatment such as Glucocorticoid and/or antibiotics. We also included studies that compared CHE combined with conventional medicine to conventional medicine alone. Studies with inadequate CHE quality control were excluded.

### 2.3. Types of Outcome Measures

*(1) Incidence of RILI*. Primary outcome was the incidence of RILI, including acute RP and RIPF. We considered the Radiation Therapy Oncology Group (RTOG) scale [39] or National Cancer Institute Common Terminology Criteria for Adverse Events (NCI-CTCAE) Version 4.03 scale [40] for the clinical grading of RILI (Table 1).

*(2) Clinical Effectiveness*. Total effective rate and remission rate of RILI patients were assessed according to Union for International Cancer Control (UICC) criteria [41].

*Complete Remission (CR)*. The patients felt completely free from all symptoms, with normal results in X-ray or Computed Tomography (CT) examination for more than 4 weeks.

*Partial Remission (PR)*. Symptoms alleviated obviously, with shadows reduced ≥50% in X-ray or CT examination for more than 4 weeks.

*Not Cured (NC)*. Compared with before treatment, symptoms did not alleviate with shadows or fibrosis in X-ray or CT examination.

(1) $$\begin{array}{c} \text{Total Effective Rate = CR + PR.} \end{array}$$

*(3) Quality of Life (Qol)*. Patient's Qol was measured before and after radiotherapy using the Karnofsky performance status (KPS) scale. An increase of 10 points or more on the KPS score was considered improvement.

*(4) Clinical Symptoms and Signs*. Duration of fever, cough, asthma exacerbation, and colored sputum was evaluated.

*(5) Inflammatory Cytokines*. Interleukin-6 (IL-6), TGF-*β*, and TNF-*α* cytokines in plasma were evaluated.

*(6) Thymus Dependent Lymphocytes (T Lymphocytes) Subgroups and Natural Killer (NK) Cells*. T lymphocytes subgroups in peripheral blood were evaluated, including CD3+ lymphocytes, CD4+ lymphocytes, and CD8+ lymphocytes. Natural killer (NK) cells were also evaluated.

*(7) Incidence of Adverse Events*. Adverse events related to CHE intervention were extracted.

### 2.4. Data Extraction and Quality Assessment

The included studies were evaluated and the relevant data were extracted by two authors (BD and CD) independently. Differences were resolved by a third party. An electronic database was established using EndNotX7 software. The improved Jadad scale was used to assess the quality of the trials, including randomization; blinding of participants, personnel, and outcome assessors; completeness of outcome data; and other threats to validity [42]. High quality is 4–7 points. Low quality is 1–3 points.

### 2.5. Data Synthesis

Meta-analysis was conducted using RevMan 5.0 (Cochrane Collaboration, UK). Dichotomous data were expressed as odds ratio (OR) and continuous data were expressed as mean difference (MD). Heterogeneity between results was assessed by *I*^2^ statistics and Cochran's *Q*-test, and *I*^2^ > 50% or *P* < 0.10 was defined as indicating heterogeneity. The fixed-effect model was used in homogeneity data merging and the random-effects model was suitable for the merging of heterogeneous data [42]. Subgroups were divided according to constituents of CHE intervention. CHE containing the same ingredients was combined into 1 subgroup. Publication bias was evaluated by visual assessment of the asymmetry of funnel plots.

## 3. Results

### 3.1. Description of Studies and Methodological Quality (Figure 1 and Table 2)

Primary searches identified 1167 references from the above databases. After duplicates, animal studies, case reports, reviews, and obvious ineligibility were removed, a total of 397 references were retrieved for text review. After assessment according to inclusion criteria and exclusion criteria, 28 studies were included [5–28]. Publication year of included studies ranging from 2006 to 2016, which in the years of 2012 and 2015 had a larger amount of studies than other years (14 studies, 55.82% cases). All the studies were conducted in mainland China. Since all included studies were assessed to be of high quality (improved Jadad score of 4–7 points), the risk of bias in this systematic review was low. All studies employed computer software or random number table for randomization. Three studies used conventional medicine as control; only one study performed double-blinding. Eleven trials followed participants for 1 to 12 months.

### 3.2. Participants

In total, 2734 participants receiving thoracic radiotherapy were included. The average size of the study was 98 participants, ranging from 40 to 232 per study. All studies included adult patients, both male and female, and 63.68% participants were male patients. The entities of patients included lung cancer (*n* = 1796 cases), esophagus cancer (*n* = 322 cases), breast cancer (*n* = 164 cases), and mediastinal malignant tumors (*n* = 33 cases). The entities of the other 419 cases were not mentioned. Eighteen studies only enrolled inpatients (*n* = 1577 cases, 57.68%), the remaining 10 studies did not report the setting (*n* = 1157 cases, 42.32%). The dose of radiotherapy varies from 30 to 76 Gy but mostly 38 to 70 Gy (15 studies). Thirteen studies used RTOG grade of RILI. Other studies used NCI-CTCAE, UICC, or other grading scales (described in references) to quantify the grade of RILI.

### 3.3. Intervention Comparisons (Table 3)

Sixteen CHE were evaluated, including 14 injections and 2 oral medicines. Shen Mai Injection (SMI) and Tan Re Qing Injection (TRQI) were most widely used in these studies. Six studies evaluated extractions from single herb or active ingredient(s). The other 22 studies evaluated extractions from Traditional Chinese medicine (TCM) compound prescriptions. The most popular herbs were* Radix Ginseng (Rubra)*/*Radix Codonopsis* (9 times).* Radix Scutellariae, Flos Lonicerae *(5 times)*, Fructus Forsythiae *(5 times), and* Rhizoma Chuanxiong *(5 times) were also popular.

Thirteen studies (*n* = 1470 cases) compared CHE to placebo or no treatment. Twelve studies (*n* = 937 cases) tested CHE combined with Glucocorticoid and/or antibiotics, compared to the same western medications for RILI management. And other 3 studies (*n* = 327 cases) tested CHE compared to Glucocorticoid and/or antibiotics. Three kinds of administration methods were employed in these 28 studies, including intravenous drip (25 studies), oral administration (2 studies), and aerosol inhalation (1 study). The duration of intervention varied from 7 days to 75 days but mostly 14 to 35 days (22 studies).

### 3.4. Preventing Effects of Interventions

#### 3.4.1. Incidence of Acute Radiation Pneumonitis (Figure 2)

Sixteen studies reported incidence of grades 1–5 acute RP. Ten CHE were evaluated and 1789 cases were included. The incidence of acute RP in CHE group was 34.76% and control group was 56.07%. Meta-analysis showed incidence of acute RP was significantly reduced in CHE group (*P* < 0.01, OR = 0.34, 95% CI [0.28–0.43]). Subgroup analysis showed all CHE reduced the incidence of acute RP, except for Elemene Injection (EI).

#### 3.4.2. Incidence of Radiation Induced Pulmonary Fibrosis (Figure 3)

Five trials (*n* = 573 cases) reported incidence of RIPF, and three CHE were evaluated. The incidence of RIPF in CHE group was 22.38%, with 44.60% in control group. Subgroup analysis showed that all CHE reduced the incidence of radiation pulmonary fibrosis (*P* < 0.01, OR = 0.35, 95% CI [0.24–0.50]).

### 3.5. Therapeutic Effects of Interventions 

#### 3.5.1. Total Effective Rate (Figure 4)

Twelve trials included 1069 cases that already developed RILI and reported curative effects of 9 CHE injections intervention. The total effective rate (TER) of CHE group was 90.91%, and control group was 71.29%. Meta-analysis showed TER of RILI patients increased significantly in CHM group (*P* < 0.01, OR = 4.44, 95% CI [3.10–6.36]). Subgroup analysis showed all CHE injections significantly increased the TER of RILI patients (*P* < 0.01 or *P* < 0.05), except for Danshen Ligustrazine Injection (DLI), Shen Qi Fu Zheng Injection (SQFZI), and Xue Bi Jing Injection (XBJI).

#### 3.5.2. Complete Remission Rate (Figure 5)

Eleven trials (1028 cases) evaluated the complete remission (CR) rate of 8 CHE injections intervention. The CR of CHE group was 52.26% and control group was 33.13%. Meta-analysis showed CR of RILI patients increased significantly in CHE group (*P* < 0.01, OR = 2.43, 95% CI [1.86–3.17]). Subgroup analysis showed DLI, Lian Bi Zhi Injection (LBZI), TRQI, and XBJI significantly increased the CR (*P* < 0.01 or *P* < 0.05).

### 3.6. Percentage of Quality of Life Improvement (Figure 6)

Eight studies reported percentage of Qol improvement. Seven CHE injections were evaluated and 719 cases were included. The percentage of Qol improvement in CHE group was 60.67%, with 33.33% in control group. Meta-analysis showed the percentage of Qol improvement increased significantly in CHE group (*P* < 0.01, OR = 3.61, 95% CI [2.58–5.06]). Subgroup analysis showed all CHE injections significantly increased percentage of Qol improvement (*P* < 0.01 or *P* < 0.05), except for EI, LBZI, and TRQI.

### 3.7. Clinical Symptoms and Signs (Supplemental Figure  1)

Two studies (*n* = 199 cases) investigated the duration of fever, cough, asthma, and colored sputum. Meta-analysis and subgroup analysis showed LBZI and TRQI significantly reduced the duration of fever individually (*P* < 0.01, MD = −1.23, 95% CI [−1.81–−0.66]). The duration of asthma and colored sputum also reduced significantly (*P* < 0.01 MD = −1.43, 95% CI [−2.50–−0.36]; *P* < 0.05, MD = −1.99, 95% CI [−3.71–−0.26]) (see Supplementary Material available online at https://doi.org/10.1155/2017/2141645).

### 3.8. Inflammatory and Fibrosis Cytokines (Supplemental Figure  2)

#### 3.8.1. TGF-*β* Cytokine

Four studies (*n* = 368 cases) investigated TGF-*β* cytokine in plasma after treatments of 3 CHE individually. Meta-analysis and subgroup analysis showed these CHE significantly reduced TGF-*β* cytokine in plasma individually (*P* < 0.01, MD = −3.46, 95% CI [−4.20–−2.73]).

#### 3.8.2. TNF-*α* Cytokine

Two studies (*n* = 140 cases) investigated TNF-*α* cytokine in plasma after Danshen Injcection (DI) or Ligustrazine Injection (LI) treatment. Meta-analysis and subgroup analysis showed these injections reduced TNF-*α* cytokine in plasma individually (*P* < 0.01, MD = −1.42, 95% CI [−1.60–−1.25]).

#### 3.8.3. IL-6 Cytokine

Three studies (*n* = 158 cases) investigated IL-6 cytokine in plasma after treatments of 3 CHE injections individually. Meta-analysis and subgroup analysis showed these injections significantly reduced plasma IL-6 cytokine individually (*P* < 0.01, MD = −25.17, 95% CI [−42.68–−7.66]).

### 3.9. Thymus Dependent Lymphocytes Subgroups and Natural Killer Cells (Supplemental Figure  3)

Two studies (*n* = 211 cases) investigated subset of T lymphocytes in peripheral blood after Compound Kushen Injection (CKI) or SMI treatment. Meta-analysis and subgroup analysis showed these injections significantly increased the levels of CD3+ T lymphocytes (*P* < 0.01, MD = 9.63, 95% CI [3.68–15.58]), CD4+ T lymphocytes (*P* < 0.01, MD = 10.16, 95% CI [6.58–13.74]), and NK cells (*P* < 0.05, MD = 6.25, 95% CI [5.81–6.70]), while changes of CD8+ T lymphocytes were not statistical differences.

### 3.10. Adverse Events and Publication Bias (Figure 7)

No adverse events associated with CME interventions were reported among included studies. Exploration of the funnel plot for incidence of acute RP, TER, and CR of RILI patients between CME group and control suggested almost symmetry; no significant publication bias was showed.

## 4. Discussions

Radiation is one of the main therapies for lung cancer and other malignancies, but RILI is a dose-limiting factor for radiotherapy and is sometimes life-threatening. RILI limits the effectiveness, dose, and schedule of radiotherapy and reduces the Qol of patients. At present, there is a trend emphasizing the Qol in cancer patients receiving radiotherapy in addition to its cancer-killing effects. Thus, the prevention of RILI with CHE represents a new avenue for dealing with these side effects of radiotherapy.

Early RILI manifests as acute RP, occurs between 1 and 3 months of radiation, and is characterized by the loss of type I pneumocytes, increases in capillary permeability, interstitial edema, and alveolar capillary congestion, and the accumulation of inflammatory and immune cells from peripheral blood in the alveolar space. In this stage, LBZI, SMI, and TRQI reduced the incidence of acute RP and increased TER and CR in RP patients. CKI, LI, Shen Fu Injection (SFI), and SQFZI, prevented the incidence of RP, while Ai Di Injection (ADI), DI, DLI, Shuang Huang Lian Injection (SHLI), and XBJI increased TER or CR in acute RP patients. The proinflammatory cytokines (TNF-*α*and IL-6) were reduced by DI and SFI. CKI and SMI improved T lymphocytes subgroups in peripheral blood.

Radiation induces a second wave release of profibrosis cytokines (including TGF-*β*), around 6–8 weeks after radiation. The increased TGF-*β* causes influx of fibroblasts and their conversion to myofibroblasts, thus causing lung fibrosis. This in turn causes hypoxia which induces profibrosis and proangiogenic cytokines release. The vicious cycle continues and leads to chronic lung disease [1]. In this stage, berberine, LI, and TRQI significantly reduced the incidence of RIPF. TGF-*β* decreased significantly after berberine, EI, and TRQI intervention; therefore the vicious cycle was broken.

RILI is not recorded in classic TCM books. Based on syndrome differentiation and treatment, TCM oncologists believe radiation falls under the category of heat toxin in TCM theory. CKI, TRQI, SHLI, and LBZI are extracted from herbs with clearing heat and removing toxicity efficacy. These treatments reduced the duration of fever and colored sputum and relieved pneumonia in vivo [35]. CKI have anticancer effects for various types of solid tumors, such as non-small-cell lung cancer, liver cancer, breast cancer, and so on [43, 44]. Matrine (the main chemical ingredient of CKI) and its derivatives exhibit a variety of pharmacological activities including anticancer, anti-inflammatory, and antifibrotic effects [43, 44]. They could also prevent or reduce chemotherapy- and/or radiotherapy-induced toxicity and reduce cancer induced pain [45, 46].* Radix Scutellariae, Flos Lonicerae, and Fructus Forsythiae* in TRQI and SHLI have anti-inflammatory and antioxidative properties in various models of lung injury [47–49]. Baicalin, extracted and purified from* Radix Scutellariae*, exerts an inhibitory effect on airway inflammation, and this effect may be associated with the inhibition of CC chemokine receptor 7 (CCR7) and its ligands CCL19/CCL21 [47]. Luteolin, an active flavonoid compound isolated from* Flos Lonicerae*, has a potent antifibrotic activity; this effect was mediated, at least in part, by inhibition of lung inflammation and suppression of myofibroblast differentiation as well as epithelial-to-mesenchymal transition [48]. Forsythin, an active ingredient extracted from* Fructus Forsythiae, *exerts anti-inflammatory action via suppressing LPS-induced activation of JAK-STATs and p38 MAPKs signaling and production of ROS in macrophage cells [49]. Andrographolide, from* Herba Andrographis* in LBZI, markedly hampered the activation of nuclear factor-*κ*B (NF-*κ*B) and NLRP3 inflammasome both in vivo and in vitro thus decreasing levels of TNF-*α* and IL-1*β* [50].

In TCM theory, heat toxin burns lung Yin, injures Fluid, and consumes Qi. ADI, SFI, SMI, SQFZI, and Yang Zheng Xiao Ji Capsule (YZXJC) are extracted from herbs with reinforcing Qi and nourishing Yin efficacy [36, 37].* Radix Ginseng* is a main component of these 5 CHE. A high pretreatment dose of ginseng extractions resulted in a marked attenuation of the severity of inflammatory changes in lung tissue in a mouse model of RILI and led to significant reductions in TNF-*α* and TGF-*β*1 cytokines [51]. In vivo, ginseng extractions and SFI led to significant reductions in TNF-*α* and TGF-*β*1 cytokines. This may be a key mechanism behind the preventive effects of these 4 injections on RILI [36]. Ginsenoside Rg3 a main ingredient of ginseng extractions was able to sensitize A549 and H1299 lung carcinoma cells to *γ*-radiation and significantly enhance the efficacy of radiation therapy in C57BL/6 mice bearing a Lewis lung carcinoma cell xenograft tumor [52].* Radix Astragali* in SQFZI and YZXJC is considered as potential and powerful exogenous source of antioxidants. It provides significant protection against lung injury in various models of oxidative stress-related disease [53].

Lung fibrosis falls under the category of blood stasis in TCM theory.* Radix Salviae Miltiorrhizae, Rhizoma Chuanxiong*,* Radix Curcumae, *and* Rhizoma Curcumae* promote blood circulation to remove blood stasis. Their extractions tanshinone, LI, and EI relieved RILI in vivo [38] and have antitumor effects against lung cancer [54–56]. EI also enhances the radiosensitivity of lung adenocarcinoma [57].

This review has its limitations. We only included studies published in journals. Dissertations and conference papers were not included. Only high quality (improved Jadad score ≥ 4 points) trials were included. We excluded 285 trials with low quality or insufficient information for assessing risk of bias. We only included studies about herbal extractions approved by CFDA, herbal extractions without quality control were excluded. Therefore, it may not be possible to achieve a complete summary of all existent evidence. Studies conducted in different countries, which would strengthen the results on the different scales, were lacking. Future trials should assure adequate concealment of allocation and blinding of outcome assessors. And long-term outcomes, such as PFS (Progression-Free-Survival) and OS (Overall survival), should also be evaluated.

## 5. Conclusions

From our study, we found that herbal medicine extractions intervention may have clinical effectiveness for relieving RILI and related symptoms and signs and lead to improvement of Qol. However, the evidence is not sufficient. More double-blind, multicenter, large-scale RCTs are needed to draw definitive conclusions.

## Supplementary Material

Supplemental Figure 1: Forest plot of clinical symptoms and signs (a) Duration of fever (b) Duration of asthma (c) Duration of colored sputum.
Supplemental Figure 2: Forest plot of inflammatory cytokine levels (a) TGF-*β* levels, (b) TNF-*α* levels, (c) IL-6 levels.
Supplemental Figure 3: Forest plot of comparison: (a) CD3+ thymus dependent lymphocytes levels, (b) CD4+ thymus dependent lymphocytes levels, (c) NK cell levels.

## Figures and Tables

**Figure 1:**
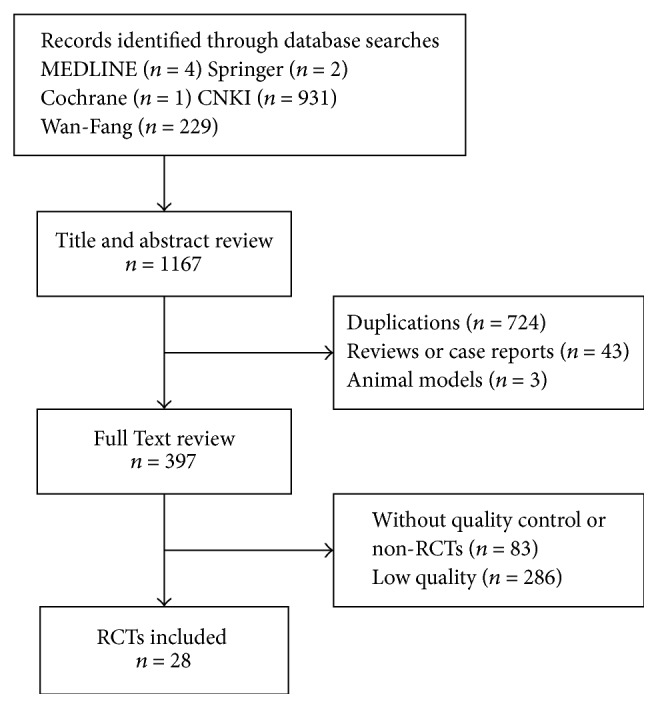
Flow chart of literature search.

**Figure 2:**
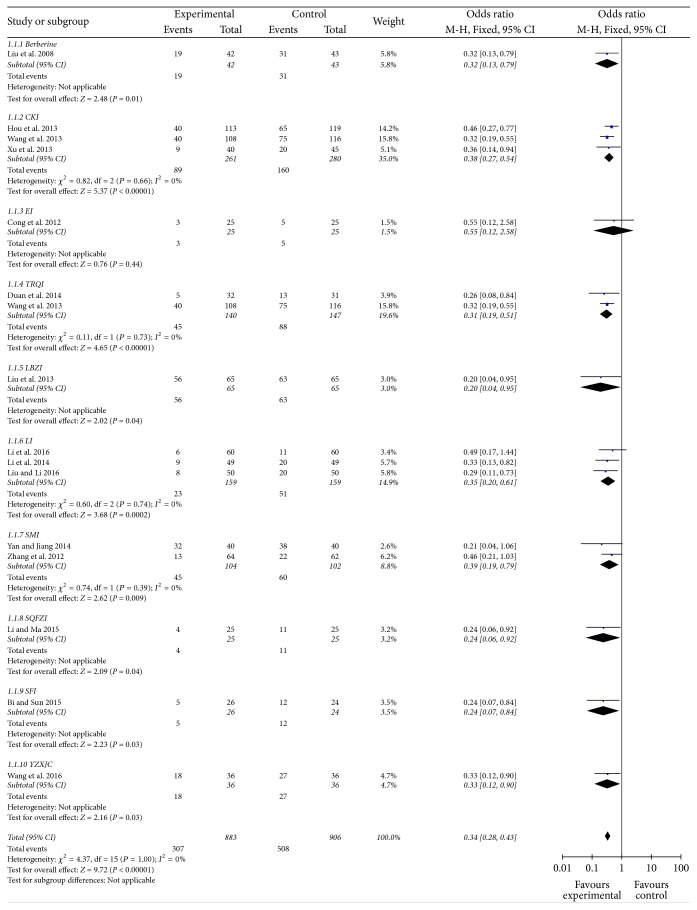
Forest plot of comparison: incidence of acute radiation pneumonitis.

**Figure 3:**
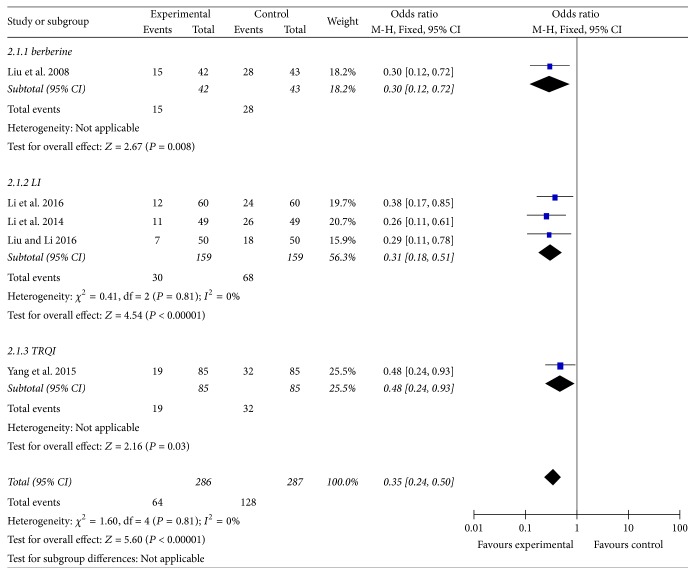
Forest plot of comparison: incidence of radiation induced pulmonary fibrosis.

**Figure 4:**
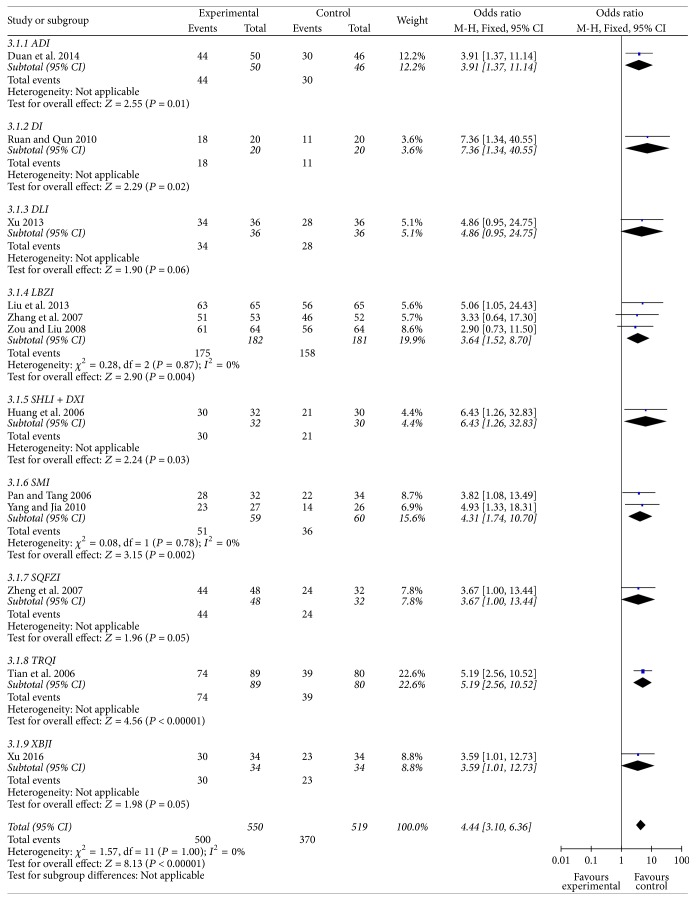
Forest plot of comparison: total effective rate of radiation induced lung injury.

**Figure 5:**
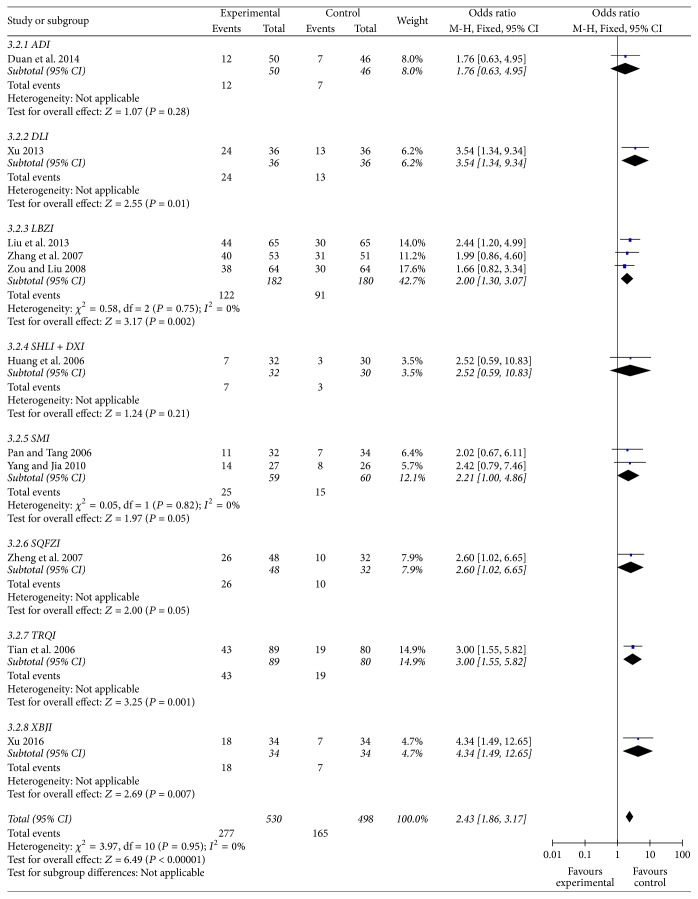
Forest plot of comparison: complete remission rate of radiation induced lung injury.

**Figure 6:**
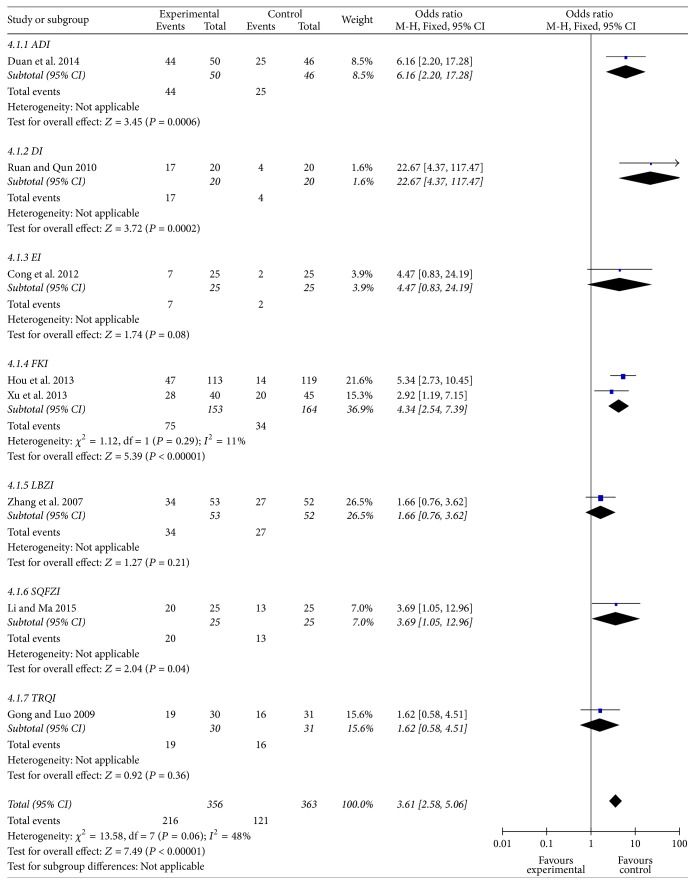
Forest plot of comparison: percentage of quality of life improvement.

**Figure 7:**
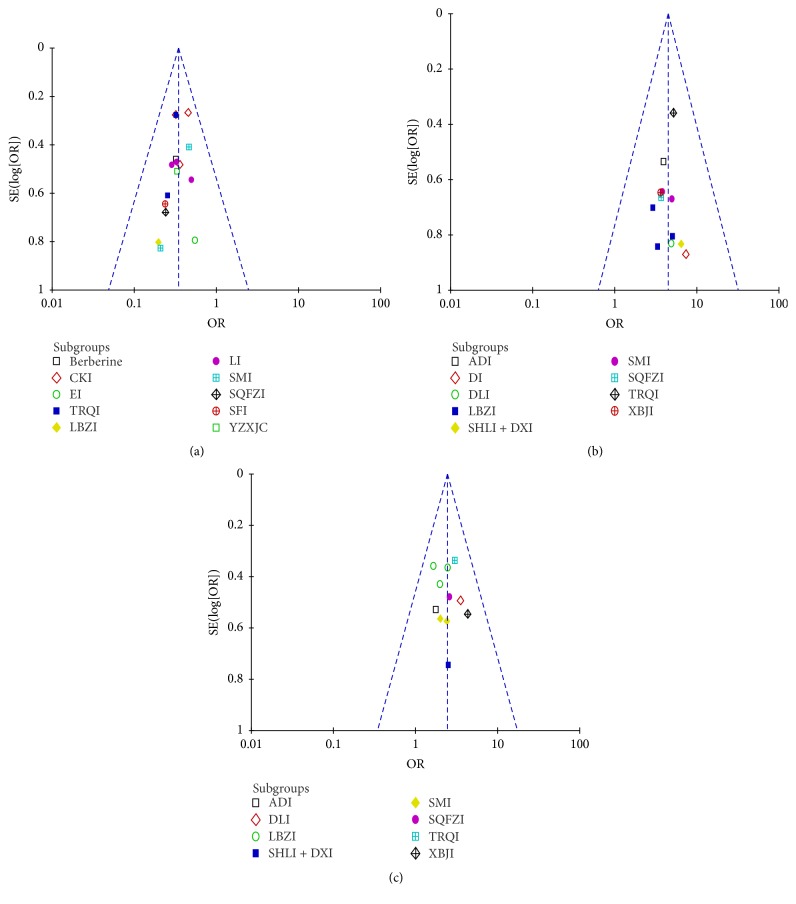
Funnel plot analysis of risk of bias. (a) Incidence of acute radiation pneumonitis. (b) Total effective rate of radiation induced lung injury. (c) Complete remission rate of radiation induced lung injury.

**Table 1:** RTOG and NCI-CTCAE Version 4.03 clinical grading scale of radiation pneumonitis.

Grade	RTOG	NCI-CTCAE
1	Mild dry cough not requiring medications	Asymptomatic; clinical or diagnostic observations only; intervention not indicated
2	Cough requiring narcotic antitussives or dyspnoea not at rest	Symptomatic; medical intervention indicated; limiting instrumental ADL
3	Severe cough not controlled by medications. Dyspnoea at rest	Severe symptoms; limiting self-care ADL; oxygen indicated
4	Continuous oxygen or assisted ventilation	Life-threatening respiratory compromise; urgent intervention indicated (e.g., tracheotomy or intubation)
5	Fatal	Death

**Table 2:** An overview of the included studies.

Study	Sample size	Mean age (year) (median/range)	% men	Dose of radiotherapy (Gy)	CHE intervention	Control	Administration of CHE	Dose of CHE	Course of CHE (d)	Grading scale	Jadad score
Bi and Sun 2015	50	58	60.0	40–65	SFI	Placebo	iv	40 mL	42 d	RTOG	4
Cong et al. 2012	50	—	82.0	60–70	EI	—	iv	400 mg	14 d *∗* 2 cycles	RTOG	4
Duan et al. 2014	63	60.2	74.6	60–66	TRQI	—	iv	20 mL	30–33 d	RTOG	4
Duan et al. 2014	96	46.2	53.1	40–76	ADI	Glucocorticoid	iv	50 mL	20 d/40 d	RTOG	4
Gong and Luo 2009	61	61.3	62.3	—	TRQI + Antibiotics, Glucocorticoid	Antibiotics, Glucocorticoid	iv	20 mL	21 d	Reference	4
Hou et al. 2013	232	60	78.0	30–70	CKI	—	iv	15–20 mL	14 d *∗* 2 cycles	RTOG	4
Huang et al. 2006	62	58.7	79.0	—	SHLI + DXI	Antibiotics, Glucocorticoid	iv	60 mg + 10–20 mL	14 d	—	4
Li and Ma 2015	50	56	66.0	—	SQFZI + Antibiotics, Glucocorticoid	Antibiotics, Glucocorticoid	iv	250 mL	21 d	RTOG	4
Li et al. 2016	120	54.36	60.0	50–66	LI	—	iv	100 mL	35–45 d	Reference	4
Li et al. 2014	98	—	59.2	50–64	LI	—	iv	80 mg	33–42 d	RTOG	4
Liu et al. 2013	130	54	56.2	—	LBZI + Antibiotics, Glucocorticoid	Antibiotics, Glucocorticoid	iv	200 mg	21 d	Reference	4
Liu and Li 2016	100	65.9	53.0	64–70	LI	—	iv	120 mg	21 d	Reference	4
Liu et al. 2008	85	65	70.6	60–70	Berberine	Placebo	p.o.	20 mg/kg	42 d	RTOG	7
Pan and Tang 2006	66	46.2	53.0	40–70	SMI + Antibiotics, Glucocorticoid	Antibiotics, Glucocorticoid	iv	50 mL	7–14 d	RTOG	4
Ruan and Qun 2010	40	—	35.0	—	DI + Antibiotics, Glucocorticoid	Antibiotics, Glucocorticoid	iv	800 mg	21 d	Reference	4
Tian et al. 2006	169	32–78	58.0	—	TRQI	Antibiotics, Glucocorticoid	iv	20 mL	15 d	RTOG	4
Xu 2013	72	55.7	54.2	—	DLI + Antibiotics, Glucocorticoid	Antibiotics, Glucocorticoid	iv	20 mL	14 d	Reference	4
Xu 2016	68	47.1	57.4	—	XBJI + Antibiotics, Glucocorticoid	Antibiotics, Glucocorticoid	iv	200 mL	14 d	—	4
Xu et al. 2013	85	45	56.5	38–70	CKI	—	iv	30 mL	19–35 d	NCI-CTCAE	4
Wang et al. 2013	224	60	79.9	60–70	CKI	—	iv	15 mL	14 d	RTOG	4
Wang et al. 2016	72	66	61.1	60–66	YZXJC	—	p.o.	4.68 g	72–75 d	NCI-CTCAE	4
Yan and Jiang 2014	80	57	76.3	60–65	SMI	—	Aerosol inhalation	20 mL	35 d	RTOG	4
Yang and Jia 2010	53	59.5	60.4	40–76	SMI + Glucocorticoid	Glucocorticoid	iv	30 mL	28 d	UICC	4
Yang et al. 2015	170	61.2	60.6	56–64	TRQI	—	iv	20 mL	28 d	RTOG	4
Zhang et al. 2007	104	56.9	55.8	39–65	LBZI + Antibiotics, Glucocorticoid	Antibiotics, Glucocorticoid	iv	400 mg	21 d	Reference	4
Zhang et al. 2012	126	57.6	68.3	60–72	SMI	—	iv	50 mL	With radiotherapy	RTOG	4
Zheng et al. 2007	80	61.3	66.3	40–60	SQFZI + Antibiotics, Glucocorticoid	Antibiotic, Glucocorticoid	iv	250 mL	14 d	RTOG	4
Zou and Liu 2008	128	55	51.6	—	LBZI + Antibiotics, Glucocorticoid	Antibiotics, Glucocorticoid	iv	400 mg	21 d	Reference	4

**Table 3:** Constituents of included Chinese herbal extractions.

Chinese herbal extraction	Abbreviation	Medicinal herbs
Ai Di Injection	ADI	*Radix Ginseng, Radix Astragali, Radix Et Caulis Acanthopanacis Senticosi, Mylabris*
Berberine	Berberine	*Rhizoma Coptidis*
Compound Kushen Injection	CKI	*Radix Sophorae Flavescentis, Rhizoma Hterosmilacis*
Danshen Injection	DI	*Radix Salviae Miltiorrhizae *
Danshen Ligustrazine Injection	DLI	*Radix Salviae Miltiorrhizae, Rhizoma Chuanxiong*
Dan Xiang Injection	DXI	*Radix Salviae Miltiorrhizae, Lignum Dalbergiae Odoriferae*
Elemene Injection	EI	*Radix Curcumae/Rhizoma Curcumae*
Lian Bi Zhi Injection	LBZI	*Herba Andrographis *
Ligustrazine Injection	LI	*Rhizoma Chuanxiong*
Shen Fu Injection	SFI	*Radix Ginseng Rubra, Radix Aconiti Lateralis Preparata *
Shuang Huang Lian Injection	SHLI	*Fructus Forsythiae, Flos Lonicerae, Radix Scutellariae *
Shen Mai Injection	SMI	*Radix Ginseng, Radix Ophiopogonis*
Shen Qi Fu Zheng Injection	SQFZI	*Radix Codonopsis, Radix Astragali*
Tan Re Qing Injection	TRQI	*Radix Scutellariae, Fel ursi, Cornu gorais, Flos Lonicerae, Fructus Forsythiae *
Xue Bi Jing Injection	XBJI	*Flos Carthami, Radix Paeoniae Rubra, Rhizoma Chuanxiong, Radix Salviae Miltiorrhizae, Radix Angelicae Sinensis*
Yang Zheng Xiao Ji Capsule	YZXJC	*Radix Astragali, Fructus Ligustri Lucidi, Radix Ginseng, Rhizoma Curcumae, Ganoderma, *and so on
