# Supplementary material for: Chinese Herbal Extractions for Relieving Radiation Induced Lung Injury: A Systematic Review and Meta-Analysis
Source: Evid Based Complement Alternat Med. 2017 Mar 29;2017:2141645. doi: 10.1155/2017/2141645 (PMC5390604; doi:10.1155/2017/2141645)
Supplement: Supplementary file 1 — Supplemental Figure 1: Forest plot of clinical symptoms and signs (a) Duration of fever (b) Duration of asthma (c) Duration of colored sputum. Supplemental Figure 2: Forest plot of inflammatory cytokine levels (a) TGF-β levels, (b) TNF-α levels, (c) IL-6 levels. Supplemental Figure 3: Forest plot of comparison: (a) CD3+ thymus dependent lymphocytes levels, (b) CD4+ thymus dependent lymphocytes levels, (c) NK cell levels. [file 2141645.f1.zip › s1_ECAM_1883228.pdf]

Study or Subgroup	Experimental Mean	Experimental SD	Experimental Total	Control Mean	Control SD	Control Total	Weight	Mean Difference IV, Random, 95% CI
**5.1.1 LBZI**								
Liu Guifang2013	3.8	1.1	65	5.3	1.5	65	54.6%	-1.50 [-1.95, -1.05]
**Subtotal (95% CI)**			**65**			**65**	**54.6%**	**-1.50 [-1.95, -1.05]**
Heterogeneity: Not applicable								
Test for overall effect: Z = 6.50 (P < 0.00001)								
**5.1.2 TRQI**								
Tian Chuntao2006	2.96	1.47	89	3.87	2.21	80	45.4%	-0.91 [-1.48, -0.34]
**Subtotal (95% CI)**			**89**			**80**	**45.4%**	**-0.91 [-1.48, -0.34]**
Heterogeneity: Not applicable								
Test for overall effect: Z = 3.12 (P = 0.002)								
**Total (95% CI)**			**154**			**145**	**100.0%**	**-1.23 [-1.81, -0.66]**
Heterogeneity: $Tau^2 = 0.10$; $Chi^2 = 2.51$, df = 1 (P = 0.11); $I^2 = 60\%$								
Test for overall effect: Z = 4.19 (P < 0.0001)								

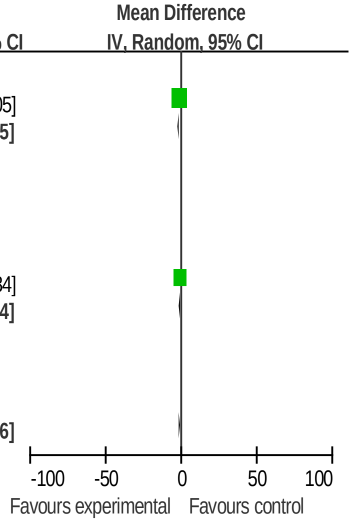

(a)

Study or Subgroup	Experimental Mean	Experimental SD	Experimental Total	Control Mean	Control SD	Control Total	Weight	Mean Difference IV, Random, 95% CI
**5.2.1 LBZI**								
Liu Guifang2013	4	1.1	65	4.9	1.9	65	51.3%	-0.90 [-1.43, -0.37]
**Subtotal (95% CI)**			**65**			**65**	**51.3%**	**-0.90 [-1.43, -0.37]**
Heterogeneity: Not applicable								
Test for overall effect: Z = 3.31 (P = 0.0009)								
**5.2.2 TRQI**								
Tian Chuntao2006	4.87	1.29	89	6.86	2.62	80	48.7%	-1.99 [-2.62, -1.36]
**Subtotal (95% CI)**			**89**			**80**	**48.7%**	**-1.99 [-2.62, -1.36]**
Heterogeneity: Not applicable								
Test for overall effect: Z = 6.16 (P < 0.00001)								
**Total (95% CI)**			**154**			**145**	**100.0%**	**-1.43 [-2.50, -0.36]**
Heterogeneity: $Tau^2 = 0.50$; $Chi^2 = 6.65$, df = 1 (P = 0.010); $I^2 = 85\%$								
Test for overall effect: Z = 2.63 (P = 0.009)								

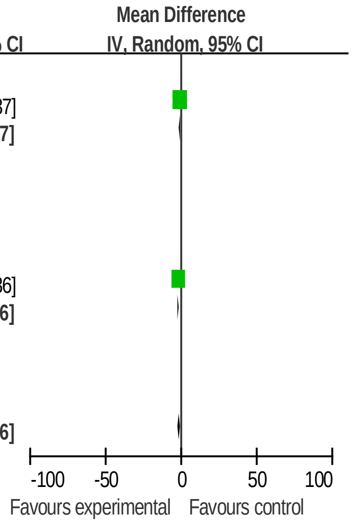

(b)

Study or Subgroup	Experimental Mean	Experimental SD	Experimental Total	Control Mean	Control SD	Control Total	Weight	Mean Difference IV, Random, 95% CI
**5.3.1 LBZI**								
Liu Guifang2013	11.2	2.5	65	14.1	2.6	65	48.0%	-2.90 [-3.78, -2.02]
**Subtotal (95% CI)**			**65**			**65**	**48.0%**	**-2.90 [-3.78, -2.02]**
Heterogeneity: Not applicable								
Test for overall effect: Z = 6.48 (P < 0.00001)								
**5.3.2 TRQI**								
Tian Chuntao2006	7.36	1.29	89	8.5	2.18	80	52.0%	-1.14 [-1.69, -0.59]
**Subtotal (95% CI)**			**89**			**80**	**52.0%**	**-1.14 [-1.69, -0.59]**
Heterogeneity: Not applicable								
Test for overall effect: Z = 4.08 (P < 0.0001)								
**Total (95% CI)**			**154**			**145**	**100.0%**	**-1.99 [-3.71, -0.26]**
Heterogeneity: $Tau^2 = 1.41$; $Chi^2 = 11.13$, df = 1 (P = 0.0008); $I^2 = 91\%$								
Test for overall effect: Z = 2.26 (P = 0.02)								

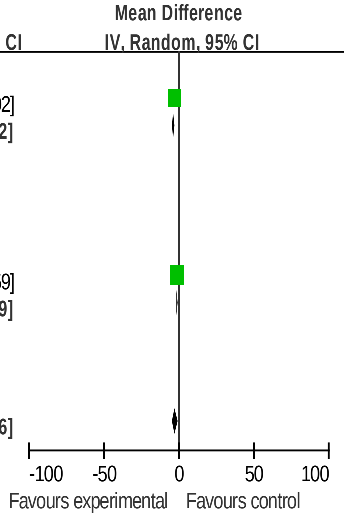

（c）

Supplementary Materials 1: Forest plot of clinical symptoms and signs
(a) Duration of fever (b) Duration of asthma (c) Duration of colored sputum
