# Supplementary material for: Chinese Herbal Extractions for Relieving Radiation Induced Lung Injury: A Systematic Review and Meta-Analysis
Source: Evid Based Complement Alternat Med. 2017 Mar 29;2017:2141645. doi: 10.1155/2017/2141645 (PMC5390604; doi:10.1155/2017/2141645)
Supplement: Supplementary file 1 — Supplemental Figure 1: Forest plot of clinical symptoms and signs (a) Duration of fever (b) Duration of asthma (c) Duration of colored sputum. Supplemental Figure 2: Forest plot of inflammatory cytokine levels (a) TGF-β levels, (b) TNF-α levels, (c) IL-6 levels. Supplemental Figure 3: Forest plot of comparison: (a) CD3+ thymus dependent lymphocytes levels, (b) CD4+ thymus dependent lymphocytes levels, (c) NK cell levels. [file 2141645.f1.zip › s2_ECAM_1883229.pdf]

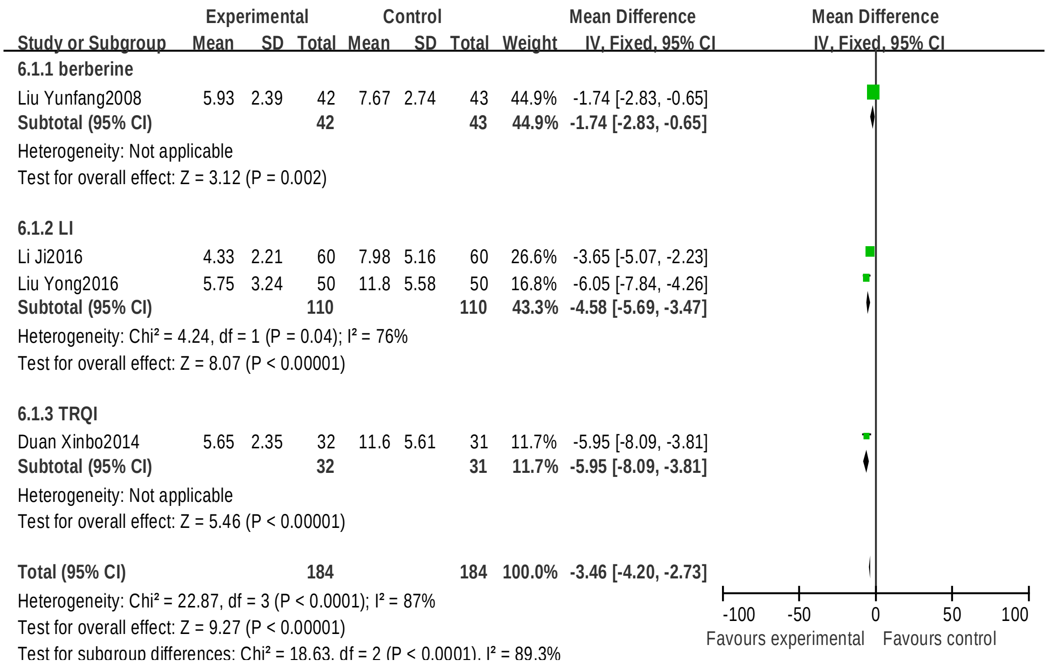

(a)

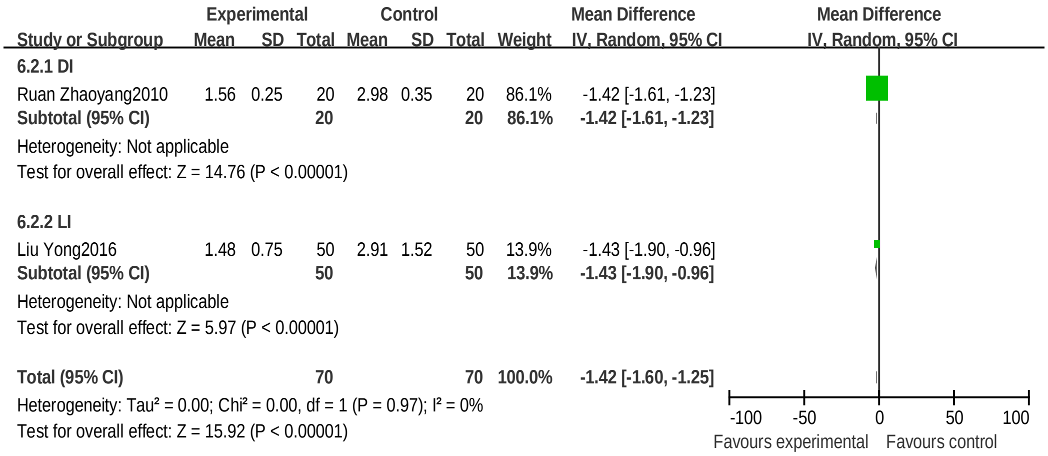

(b)

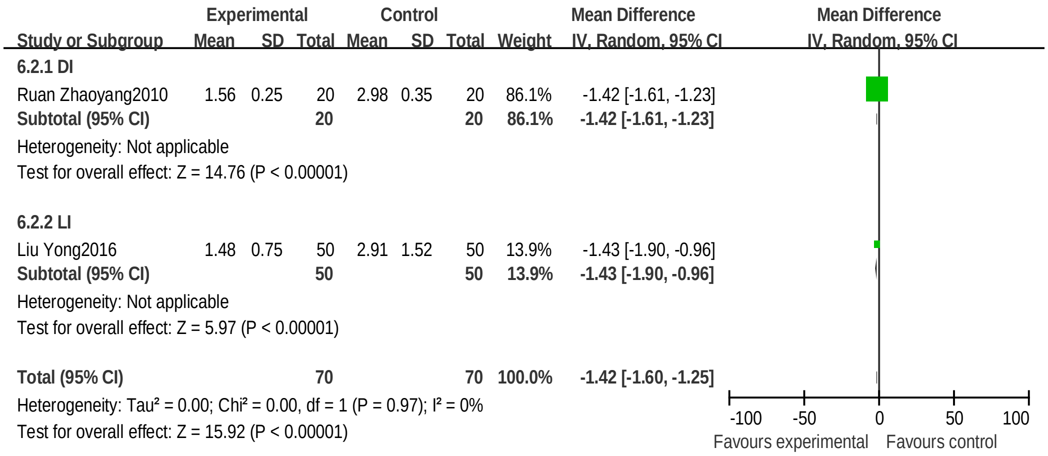

(c)

Supplementary Materials 2: Forest plot of inflammatory cytokine levels
(a) TGF-β levels, (b) TNF-α levels, (c)IL-6 levels
